# Supplementary material for: Assessment of the targeted effect of Sijunzi decoction on the colorectal cancer microenvironment via the ESTIMATE algorithm
Source: PLoS One. 2022 Mar 18;17(3):e0264720. doi: 10.1371/journal.pone.0264720 (PMC8932555; doi:10.1371/journal.pone.0264720)
Supplement: S1 Table — (DOCX) [file pone.0264720.s001.docx]

S1 Table. Basic information of some active components of SJZD

| Mol ID | Chemical Component | OB/% | DL | Herb |
| --- | --- | --- | --- | --- |
| MOL000273 | (2R)-2-[(3S,5R,10S,13R,14R,16R,17R)-3,16-dihydroxy-4,4,10,13,14-pentamethyl-2,3,5,6,12,15,16,17-octahydro-1H-cyclopenta[a]phenanthren-17-yl]-6-methylhept-5-enoic acid | 30.93 | 0.81 | Poria Cocos(Schw.) Wolf. |
| MOL000275 | trametenolic acid | 38.71 | 0.80 |  |
| MOL000276 | 7,9(11)-dehydropachymic acid | 35.11 | 0.81 |  |
| MOL000279 | Cerevisterol | 37.96 | 0.77 |  |
| MOL000280 | (2R)-2-[(3S,5R,10S,13R,14R,16R,17R)-3,16-dihydroxy-4,4,10,13,14-pentamethyl-2,3,5,6,12,15,16,17-octahydro-1H-cyclopenta[a]phenanthren-17-yl]-5-isopropyl-hex-5-enoic acid | 31.07 | 0.82 |  |
| MOL000282 | ergosta-7,22E-dien-3beta-ol | 43.51 | 0.72 |  |
| MOL000283 | Ergosterol peroxide | 40.36 | 0.81 |  |
| MOL000285 | (2R)-2-[(5R,10S,13R,14R,16R,17R)-16-hydroxy-3-keto-4,4,10,13,14-pentamethyl-1,2,5,6,12,15,16,17-octahydrocyclopenta[a]phenanthren-17-yl]-5-isopropyl-hex-5-enoicš | 38.26 | 0.82 |  |
| MOL000287 | 3beta-Hydroxy-24-methylene-8-lanostene-21-oic acid | 38.70 | 0.81 |  |
| MOL000289 | pachymic acid | 33.63 | 0.81 |  |
| MOL000290 | Poricoic acid A | 30.61 | 0.76 |  |
| MOL000291 | Poricoic acid B | 30.52 | 0.75 |  |
| MOL000292 | poricoic acid C | 38.15 | 0.75 |  |
| MOL000296 | hederagenin | 36.91 | 0.75 |  |
| MOL000300 | dehydroeburicoic acid | 44.17 | 0.83 |  |
| MOL001484 | Inermine | 75.18 | 0.54 | licorice |
| MOL001792 | DFV | 32.76 | 0.18 |  |
| MOL000211 | Mairin | 55.38 | 0.78 |  |
| MOL002311 | Glycyrol | 90.78 | 0.67 |  |
| MOL000239 | [Jaranol](https://tcmspw.com/molecule.php?qn=239) | 50.83 | 0.29 |  |
| MOL002565 | [Medicarpin](https://tcmspw.com/molecule.php?qn=2565) | 49.22 | 0.34 |  |
| MOL000354 | [isorhamnetin](https://tcmspw.com/molecule.php?qn=354) | 49.6 | 0.31 |  |
| MOL000359 | [sitosterol](https://tcmspw.com/molecule.php?qn=359) | 36.91 | 0.75 |  |
| MOL003656 | [Lupiwighteone](https://tcmspw.com/molecule.php?qn=3656) | 51.64 | 0.37 |  |
| MOL003896 | [7-Methoxy-2-methyl isoflavone](https://tcmspw.com/molecule.php?qn=3896) | 42.56 | 0.2 |  |
| MOL000392 | [formononetin](https://tcmspw.com/molecule.php?qn=392) | 69.67 | 0.21 |  |
| MOL000417 | [Calycosin](https://tcmspw.com/molecule.php?qn=417) | 47.75 | 0.24 |  |
| MOL000422 | [kaempferol](https://tcmspw.com/molecule.php?qn=422) | 41.88 | 0.24 |  |
| MOL004328 | [naringenin](https://tcmspw.com/molecule.php?qn=4328) | 59.29 | 0.21 |  |
| MOL004805 | [(2S)-2-[4-hydroxy-3-(3-methylbut-2-enyl)phenyl]-8,8-dimethyl-2,3-dihydropyrano[2,3-f]chromen-4-one](https://tcmspw.com/molecule.php?qn=4805) | 31.79 | 0.72 |  |
| MOL004806 | [euchrenone](https://tcmspw.com/molecule.php?qn=4806) | 30.29 | 0.57 |  |
| MOL004808 | [glyasperin B](https://tcmspw.com/molecule.php?qn=4808) | 65.22 | 0.44 |  |
| MOL004810 | [glyasperin F](https://tcmspw.com/molecule.php?qn=4810) | 75.84 | 0.54 |  |
| MOL004811 | [Glyasperin C](https://tcmspw.com/molecule.php?qn=4811) | 45.56 | 0.4 |  |
| MOL004814 | [Isotrifoliol](https://tcmspw.com/molecule.php?qn=4814) | 31.94 | 0.42 |  |
| MOL004815 | [(E)-1-(2,4-dihydroxyphenyl)-3-(2,2-dimethylchromen-6-yl)prop-2-en-1-one](https://tcmspw.com/molecule.php?qn=4815) | 39.62 | 0.35 |  |
| MOL004820 | [kanzonols W](https://tcmspw.com/molecule.php?qn=4820) | 50.48 | 0.52 |  |
| MOL004824 | [(2S)-6-(2,4-dihydroxyphenyl)-2-(2-hydroxypropan-2-yl)-4-methoxy-2,3-dihydrofuro[3,2-g]chromen-7-one](https://tcmspw.com/molecule.php?qn=4824) | 60.25 | 0.63 |  |
| MOL004827 | [Semilicoisoflavone B](https://tcmspw.com/molecule.php?qn=4827) | 48.78 | 0.55 |  |
| MOL004828 | [Glepidotin A](https://tcmspw.com/molecule.php?qn=4828) | 44.72 | 0.35 |  |
| MOL004829 | [Glepidotin B](https://tcmspw.com/molecule.php?qn=4829) | 64.46 | 0.34 |  |
| MOL004833 | [Phaseolinisoflavan](https://tcmspw.com/molecule.php?qn=4833) | 32.01 | 0.45 |  |
| MOL004835 | [Glypallichalcone](https://tcmspw.com/molecule.php?qn=4835) | 61.6 | 0.19 |  |
| MOL004838 | [8-(6-hydroxy-2-benzofuranyl)-2,2-dimethyl-5-chromenol](https://tcmspw.com/molecule.php?qn=4838) | 58.44 | 0.38 |  |
| MOL004841 | [Licochalcone B](https://tcmspw.com/molecule.php?qn=4841) | 76.76 | 0.19 |  |
| MOL004848 | [licochalcone G](https://tcmspw.com/molecule.php?qn=4848) | 49.25 | 0.32 |  |
| MOL004849 | [3-(2,4-dihydroxyphenyl)-8-(1,1-dimethylprop-2-enyl)-7-hydroxy-5-methoxy-coumarin](https://tcmspw.com/molecule.php?qn=4849) | 59.62 | 0.43 |  |
| MOL004855 | [Licoricone](https://tcmspw.com/molecule.php?qn=4855) | 63.58 | 0.47 |  |
| MOL004856 | [Gancaonin A](https://tcmspw.com/molecule.php?qn=4856) | 51.08 | 0.4 |  |
| MOL004857 | [Gancaonin B](https://tcmspw.com/molecule.php?qn=4857) | 48.79 | 0.45 |  |
| MOL004863 | [3-(3,4-dihydroxyphenyl)-5,7-dihydroxy-8-(3-methylbut-2-enyl)chromone](https://tcmspw.com/molecule.php?qn=4863) | 66.37 | 0.41 |  |
| MOL004864 | [5,7-dihydroxy-3-(4-methoxyphenyl)-8-(3-methylbut-2-enyl)chromone](https://tcmspw.com/molecule.php?qn=4864) | 30.49 | 0.41 |  |
| MOL004866 | [2-(3,4-dihydroxyphenyl)-5,7-dihydroxy-6-(3-methylbut-2-enyl)chromone](https://tcmspw.com/molecule.php?qn=4866) | 44.15 | 0.41 |  |
| MOL004879 | [Glycyrin](https://tcmspw.com/molecule.php?qn=4879) | 52.61 | 0.47 |  |
| MOL004882 | [Licocoumarone](https://tcmspw.com/molecule.php?qn=4882) | 33.21 | 0.36 |  |
| MOL004883 | [Licoisoflavone](https://tcmspw.com/molecule.php?qn=4883) | 41.61 | 0.42 |  |
| MOL004884 | [Licoisoflavone B](https://tcmspw.com/molecule.php?qn=4884) | 38.93 | 0.55 |  |
| MOL004885 | [licoisoflavanone](https://tcmspw.com/molecule.php?qn=4885) | 52.47 | 0.54 |  |
| MOL004891 | [shinpterocarpin](https://tcmspw.com/molecule.php?qn=4891) | 80.3 | 0.73 |  |
| MOL004898 | [(E)-3-[3,4-dihydroxy-5-(3-methylbut-2-enyl)phenyl]-1-(2,4-dihydroxyphenyl)prop-2-en-1-one](https://tcmspw.com/molecule.php?qn=4898) | 46.27 | 0.31 |  |
| MOL004903 | [liquiritin](https://tcmspw.com/molecule.php?qn=4903) | 65.69 | 0.74 |  |
| MOL004904 | [licopyranocoumarin](https://tcmspw.com/molecule.php?qn=4904) | 80.36 | 0.65 |  |
| MOL004907 | [Glyzaglabrin](https://tcmspw.com/molecule.php?qn=4907) | 61.07 | 0.35 |  |
| MOL004908 | [Glabridin](https://tcmspw.com/molecule.php?qn=4908) | 53.25 | 0.47 |  |
| MOL004910 | [Glabranin](https://tcmspw.com/molecule.php?qn=4910) | 52.9 | 0.31 |  |
| MOL004911 | [Glabrene](https://tcmspw.com/molecule.php?qn=4911) | 46.27 | 0.44 |  |
| MOL004912 | [Glabrone](https://tcmspw.com/molecule.php?qn=4912) | 52.51 | 0.5 |  |
| MOL004913 | [1,3-dihydroxy-9-methoxy-6-benzofurano[3,2-c]chromenone](https://tcmspw.com/molecule.php?qn=4913) | 48.14 | 0.43 |  |
| MOL004914 | [1,3-dihydroxy-8,9-dimethoxy-6-benzofurano[3,2-c]chromenone](https://tcmspw.com/molecule.php?qn=4914) | 62.9 | 0.53 |  |
| MOL004915 | [Eurycarpin A](https://tcmspw.com/molecule.php?qn=4915) | 43.28 | 0.37 |  |
| MOL004924 | [(-)-Medicocarpin](https://tcmspw.com/molecule.php?qn=4924) | 40.99 | 0.95 |  |
| MOL004935 | [Sigmoidin-B](https://tcmspw.com/molecule.php?qn=4935) | 34.88 | 0.41 |  |
| MOL004941 | [(2R)-7-hydroxy-2-(4-hydroxyphenyl)chroman-4-one](https://tcmspw.com/molecule.php?qn=4941) | 71.12 | 0.18 |  |
| MOL004945 | [(2S)-7-hydroxy-2-(4-hydroxyphenyl)-8-(3-methylbut-2-enyl)chroman-4-one](https://tcmspw.com/molecule.php?qn=4945) | 36.57 | 0.32 |  |
| MOL004948 | [Isoglycyrol](https://tcmspw.com/molecule.php?qn=4948) | 44.7 | 0.84 |  |
| MOL004949 | [Isolicoflavonol](https://tcmspw.com/molecule.php?qn=4949) | 45.17 | 0.42 |  |
| MOL004957 | [HMO](https://tcmspw.com/molecule.php?qn=4957) | 38.37 | 0.21 |  |
| MOL004959 | [1-Methoxyphaseollidin](https://tcmspw.com/molecule.php?qn=4959) | 69.98 | 0.64 |  |
| MOL004961 | [Quercetin der.](https://tcmspw.com/molecule.php?qn=4961) | 46.45 | 0.33 |  |
| MOL004966 | [3'-Hydroxy-4'-O-Methylglabridin](https://tcmspw.com/molecule.php?qn=4966) | 43.71 | 0.57 |  |
| MOL000497 | [licochalcone a](https://tcmspw.com/molecule.php?qn=497) | 40.79 | 0.29 |  |
| MOL004974 | [3'-Methoxyglabridin](https://tcmspw.com/molecule.php?qn=4974) | 46.16 | 0.57 |  |
| MOL004978 | [2-[(3R)-8,8-dimethyl-3,4-dihydro-2H-pyrano[6,5-f]chromen-3-yl]-5-methoxyphenol](https://tcmspw.com/molecule.php?qn=4978) | 36.21 | 0.52 |  |
| MOL004980 | [Inflacoumarin A](https://tcmspw.com/molecule.php?qn=4980) | 39.71 | 0.33 |  |
| MOL004985 | [icos-5-enoic acid](https://tcmspw.com/molecule.php?qn=4985) | 30.7 | 0.2 |  |
| MOL004988 | [Kanzonol F](https://tcmspw.com/molecule.php?qn=4988) | 32.47 | 0.89 |  |
| MOL004989 | [6-prenylated eriodictyol](https://tcmspw.com/molecule.php?qn=4989) | 39.22 | 0.41 |  |
| MOL004990 | [7,2',4'-trihydroxy－5-methoxy-3－arylcoumarin](https://tcmspw.com/molecule.php?qn=4990) | 83.71 | 0.27 |  |
| MOL004991 | [7-Acetoxy-2-methylisoflavone](https://tcmspw.com/molecule.php?qn=4991) | 38.92 | 0.26 |  |
| MOL004993 | [8-prenylated eriodictyol](https://tcmspw.com/molecule.php?qn=4993) | 53.79 | 0.4 |  |
| MOL004996 | [gadelaidic acid](https://tcmspw.com/molecule.php?qn=4996) | 30.7 | 0.2 |  |
| MOL000500 | [Vestitol](https://tcmspw.com/molecule.php?qn=500) | 74.66 | 0.21 |  |
| MOL005000 | [Gancaonin G](https://tcmspw.com/molecule.php?qn=5000) | 60.44 | 0.39 |  |
| MOL005001 | [Gancaonin H](https://tcmspw.com/molecule.php?qn=5001) | 50.1 | 0.78 |  |
| MOL005003 | [Licoagrocarpin](https://tcmspw.com/molecule.php?qn=5003) | 58.81 | 0.58 |  |
| MOL005007 | [Glyasperins M](https://tcmspw.com/molecule.php?qn=5007) | 72.67 | 0.59 |  |
| MOL005008 | [Glycyrrhiza flavonol A](https://tcmspw.com/molecule.php?qn=5008) | 41.28 | 0.6 |  |
| MOL005012 | [Licoagroisoflavone](https://tcmspw.com/molecule.php?qn=5012) | 57.28 | 0.49 |  |
| MOL005016 | [Odoratin](https://tcmspw.com/molecule.php?qn=5016) | 49.95 | 0.3 |  |
| MOL005017 | [Phaseol](https://tcmspw.com/molecule.php?qn=5017) | 78.77 | 0.58 |  |
| MOL005018 | [Xambioona](https://tcmspw.com/molecule.php?qn=5018) | 54.85 | 0.87 |  |
| MOL005020 | [dehydroglyasperins C](https://tcmspw.com/molecule.php?qn=5020) | 53.82 | 0.37 |  |
| MOL000098 | [quercetin](https://tcmspw.com/molecule.php?qn=98) | 46.43 | 0.28 |  |
| MOL002879 | [Diop](https://tcmspw.com/molecule.php?qn=2879) | 43.59 | 0.39 | Panax Ginseng C. A. Mey. |
| MOL000449 | [Stigmasterol](https://tcmspw.com/molecule.php?qn=449) | 43.83 | 0.76 |  |
| MOL000358 | [beta-sitosterol](https://tcmspw.com/molecule.php?qn=358) | 36.91 | 0.75 |  |
| MOL003648 | [Inermin](https://tcmspw.com/molecule.php?qn=3648) | 65.83 | 0.54 |  |
| MOL000422 | [kaempferol](https://tcmspw.com/molecule.php?qn=422) | 41.88 | 0.24 |  |
| MOL004492 | [Chrysanthemaxanthin](https://tcmspw.com/molecule.php?qn=4492) | 38.72 | 0.58 |  |
| MOL005308 | [Aposiopolamine](https://tcmspw.com/molecule.php?qn=5308) | 66.65 | 0.22 |  |
| MOL005317 | [Deoxyharringtonine](https://tcmspw.com/molecule.php?qn=5317) | 39.27 | 0.81 |  |
| MOL005318 | [Dianthramine](https://tcmspw.com/molecule.php?qn=5318) | 40.45 | 0.2 |  |
| MOL005320 | [arachidonate](https://tcmspw.com/molecule.php?qn=5320) | 45.57 | 0.2 |  |
| MOL005321 | [Frutinone A](https://tcmspw.com/molecule.php?qn=5321) | 65.9 | 0.34 |  |
| MOL005344 | [ginsenoside rh2](https://tcmspw.com/molecule.php?qn=5344) | 36.32 | 0.56 |  |
| MOL005348 | [Ginsenoside-Rh4_qt](https://tcmspw.com/molecule.php?qn=5348) | 31.11 | 0.78 |  |
| MOL005356 | [Girinimbin](https://tcmspw.com/molecule.php?qn=5356) | 61.22 | 0.31 |  |
| MOL005376 | [Panaxadiol](https://tcmspw.com/molecule.php?qn=5376) | 33.09 | 0.79 |  |
| MOL005384 | [suchilactone](https://tcmspw.com/molecule.php?qn=5384) | 57.52 | 0.56 |  |
| MOL005399 | [alexandrin_qt](https://tcmspw.com/molecule.php?qn=5399) | 36.91 | 0.75 |  |
| MOL000787 | [Fumarine](https://tcmspw.com/molecule.php?qn=787) | 59.26 | 0.83 |  |
| MOL000022 | [14-acetyl-12-senecioyl-2E,8Z,10E-atractylentriol](https://tcmspw.com/molecule.php?qn=22) | 63.37 | 0.3 | Atractylodes Macrocephala Koidz. |
| MOL000033 | [(3S,8S,9S,10R,13R,14S,17R)-10,13-dimethyl-17-[(2R,5S)-5-propan-2-yloctan-2-yl]-2,3,4,7,8,9,11,12,14,15,16,17-dodecahydro-1H-cyclopenta[a]phenanthren-3-ol](https://tcmspw.com/molecule.php?qn=33) | 36.23 | 0.78 |  |
| MOL000049 | [3β-acetoxyatractylone](https://tcmspw.com/molecule.php?qn=49) | 54.07 | 0.22 |  |
| MOL000072 | [8β-ethoxy atractylenolide Ⅲ](https://tcmspw.com/molecule.php?qn=72) | 35.95 | 0.21 |  |
